# Boscisucrophage: A Natural SGLT1/2 Inhibitor From *Boscia senegalensis* for Managing Type 2 Diabetes

**DOI:** 10.1002/fsn3.71621

**Published:** 2026-03-08

**Authors:** Bruno Eto, Amal Elrherabi, Fahd A. Nasr, Joe Miantezila Basilua, Ahlam Outman, Rosette Christelle Ndjip, Mohamed Bouhrim, Mohammed Al‐zharani, Ashraf Ahmed Qurtam, Brahim Ibet, Bernard Gressier, Jehan‐François Desjeux

**Affiliations:** ^1^ Laboratories TBC, Laboratory of Pharmacology, Pharmacokinetics and Clinical Pharmacy, Faculty of Pharmacy University of Lille Lille France; ^2^ Pharmacology Unit, Laboratory of Bioresources, Biotechnology, Ethnopharmacology and Health, Faculty of Medicine and Pharmacy University Mohammed 1st Oujda Morocco; ^3^ Biology Department College of Science Riyadh Saudi Arabia; ^4^ Department of Biostatistics and Mathematics, Faculty of Pharmacy Paris City University Paris France; ^5^ Chad‐China Friendship Reference Hospital Center (HATC) N'djamena Chad; ^6^ Laboratory of Pharmacology, Pharmacokinetics and Clinical Pharmacy, Faculty of Pharmacy University of Lille Lille France; ^7^ National Academy of Medicine Paris France

**Keywords:** *Boscia senegalensis*, functional symptoms, glycemia, HbA1c, oral antihyperglycemic drug resistance, type 2 diabetes

## Abstract

*Boscia senegalensis* (Pers.) Lam. Ex Poir. (Capparaceae) is an important local famine food plant in Africa and is widely exploited by healers in the Sahelian region for its seeds, which are used to reduce hyperglycemia. We studied the efficacy of the commercial dosage form of *Boscia senegalensis* , namely Boscisucrophage (BSP), in type 2 diabetes (T2DM) patients with resistance to oral antihyperglycemic drugs. The clinical benefits of BSP were in a prospective, single‐center, open‐label, single‐arm interventional study involving 43 naïve patients and 289 diabetic patients resistant to oral antidiabetic drugs. All patients received capsules containing a fixed dose of 350 mg of BSP, taken three times daily for 12 weeks. Outcomes were monitored through venous blood glucose levels, glycosylated hemoglobin (HbA1c), urine glucose excretion (UGE), aspartate aminotransferase, alanine aminotransferase, creatinine levels, and clinical examination of functional symptoms. In the clinical study, BSP significantly reduced glycemia and HbA1c levels, increased urine glucose excretion (UGE), and alleviated the side effects and functional symptoms of T2DM. Our clinical findings provide preliminary evidence supporting the potential use of BSP to reduce glycemia and HbA1c in T2DM patients resistant to oral antihyperglycemic drugs, with no significant adverse effects observed in this study. These results highlight BSP's potential as a dual SGLT1/SGLT2 inhibitor, suggesting a novel mechanism of action. However, further validation through randomized controlled trials is necessary to confirm these findings.

**Trial Registration:** National Ethics Committee (N°679/PR/PM/MSP/SE/SG/DHATC/SGH/SRH/13)

## Graphical abstract

Boscisucrophage (BSP) significantly reduced blood glucose and HbA1c, increased urine glucose excretion, and improved functional symptoms in T2DM patients resistant to oral antihyperglycemic drugs. BSP showed no adverse effects on liver or kidney function. These findings suggest BSP as a potential dual SGLT1/SGLT2 inhibitor, warranting further validation in randomized controlled trials.

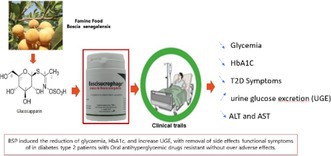

## 1 Introduction

*Boscia senegalensis* (Pers.) Lam. Ex Poir. (Capparaceae) is a tree native to the Sahel region of Africa. It is used as a local food plant, with its fruits and seeds consumed fresh or processed into various food products (Belem et al. 2017). *Boscia senegalensis* (BS) is considered a potential solution to hunger and a buffer against famine in the Sahel region due to the variety of useful products it provides. It serves multiple purposes, including as a caffeine‐free coffee alternative, livestock feed, and a remedy in both ethnoveterinary and traditional medicine. It is also utilized as a natural pest repellent for stored grains and food, incorporated into soil conservation efforts. Additionally, it functions as a food additive, a living fence, a natural coagulant for water purification, and a source of construction materials and firewood (Belem et al. 2017).

Several studies have been carried out on its composition and have shown that it is a very rich food (Kim et al. 1997). The tender roots of *Boscia senegalensis* are edible and are occasionally crushed and cooked into a porridge. The berries of *Boscia senegalensis* are edible and are commonly consumed fresh or boiled. They are also used in the preparation of sweet or alcoholic beverages and are sometimes combined with millet ( *Eleusine coracana* Gaertn.) and curdled milk to form cakes. In the Sahel region, the dried seeds of *B. senegalensis* serve as an alternative to millet or lentils ( *Lens culinaris* Medikus). Additionally, the leaves are widely foraged and used as a food condiment in soups, mixed with cereals, and serve as a vital nutritional resource during periods of food scarcity (Belem et al. 2017).

The seeds that are eaten contain almost all the amino acids and lipids and almost all the minerals, making them a food of choice. It offers products for consumption, domestic needs (pancakes, vegetables), medicinal (gastric ulcer) and agricultural uses as an insecticide to protect crops (Belem et al. 2017; Seck et al. 1993). *Boscia senegalensis* exhibits notable pharmacological properties due to its rich phytochemical profile, including saponins, alkaloids, sterols, triterpenes, flavonoids, and polyphenols. The pulp, particularly rich in polyphenols and flavonoids, shows strong reducing activity, highlighting its antioxidant potential. The plant's bioactive metabolites support its traditional use in treating infections and metabolic disorders (Kim et al. 1997). The aqueous extract of the plant also demonstrates strong hypoglycemic, hypolipidemic, and antioxidant properties. It effectively lowers blood glucose levels, improves lipid profiles by reducing triglycerides and LDL cholesterol, and mitigates oxidative stress, suggesting its potential therapeutic benefits for metabolic disorders (Seck et al. 1993). Previous studies in laboratory animals demonstrated no toxicity of BS extracts at the administered doses. Blood biochemical parameters and histological analyses of the liver, spleen, and kidney revealed no significant adverse effects, indicating that BS was well‐tolerated (Suryasa et al. 2021). Diabetes is a metabolic disorder with multiple causes, marked by persistent high blood sugar levels. It is accompanied by disruptions in carbohydrate, lipid, and protein metabolism, which result from either a deficiency in insulin secretion, impaired insulin action, or a combination of both issues (Eto 2019). Hyperglycemia contributes to both acute and long‐term degenerative metabolic complications. The global prevalence of diabetes is rising rapidly, with the number of affected individuals increasing from 108 million in 1980 to 422 million in 2014. This trend is projected to continue, with estimates suggesting that the number will reach 622 million by 2040 (Suryasa et al. 2021). The mechanisms of action of *Boscia senegalensis* dry extracts (Boscisucrophage or BSP) on blood sugar levels are not yet fully elucidated. The species appears to stimulate insulin secretion, the hormone that regulates glucose metabolism, and block the intestinal absorption of glucose by inhibiting SGLT1 (Eto 2019). Pharmacological studies in albino rabbits rendered hyperglycaemic by oral administration of D‐glucose showed that BSP exhibited optimal activity at a dose of 250 mg/kg, without causing any adverse effects. This antihyperglycemic effect was attributed to glucocapparin (Deli et al. 2019; Dongmo et al. 2017). Boscisucrophage (BSP) could be a potential source of natural anti‐diabetic molecules, provided that their efficacy and safety in humans are confirmed. The objective of this preliminary study was to assess the glycemic effects and safety of BSP as an add‐on therapy in T2DM patients with resistance to oral antihyperglycemic drugs.

## 2 Materials and Methods

### 2.1 Study Population and Ethical Considerations

This prospective, single‐arm, open‐label interventional study was conducted at the Chad‐China Friendship Hospital in N'Djamena, involving 332 patients between October 2013 and December 2022. The study received approval from the National Ethics Committee (N°679/PR/PM/MSP/SE/SG/DHATC/SGH/SRH/13). Data were collected through clinical examinations and laboratory tests. Boscisucrophage (dry aqueous extract of *Boscia senegalensis* seeds), approved under N°101.11101.0.03281 (2013), was kindly donated by Laboratoires TBC France. The plant material ( *Boscia senegalensis* ) was authenticated, and a voucher specimen (N°1344) was deposited at the Veterinary and Zoo Technical Laboratory of Farcha, Chad.

### 2.2 Study Design

This prospective, single‐arm, open‐label interventional study enrolled adult patients (> 18 years) with confirmed type 2 diabetes mellitus (T2DM) who met specific eligibility criteria. As a single‐arm study, all eligible participants received the investigational intervention (BSP) in addition to their existing glucose‐lowering therapies. No control or placebo group was included.

All participants were required to have documented poor glycemic control, defined as either HbA1c levels > 7% but ≤ 16% or fasting blood glucose ≥ 150 mg/dL at screening, despite receiving standard oral antihyperglycemic medications and/or insulin therapy for a minimum duration of 3 months. Following recommendations from the ethics committee overseeing these clinical trials, we implemented a staged enrollment strategy, prioritizing treatment‐resistant patients to first evaluate BSP's efficacy and safety as an adjunct therapy before considering treatment‐naive individuals managed solely by dietary interventions. This cautious approach ensured patient safety by initially testing the botanical supplement in subjects already stabilized on conventional antidiabetic regimens.

The exclusion criteria comprehensively addressed potential confounding factors by eliminating candidates with any history of cardiovascular compromise, hepatic impairment, or renal dysfunction. During the 90‐day intervention period, participants received standardized oral doses of BSP (350 mg capsules containing dry aqueous seed extract) administered three times daily with meals. Rigorous monitoring included serial assessments of blood glucose levels and comprehensive biochemical profiling at five predetermined time points: baseline (day −7), treatment initiation (day 0), and monthly intervals (days 30, 60, and 90). HbA1c was measured at baseline (day −1) and at days 60 and 90 only, as it reflects glycemic control over the preceding 2–3 months. Additionally, metabolic evaluation incorporated quantitative analysis of 24‐h urinary glucose excretion (10 mL aliquots collected at days −1, 2, 15, and 30). To ensure protocol adherence and patient safety, structured follow‐up visits occurred monthly, allowing for close clinical supervision and documentation of treatment outcomes (Table 1).

**TABLE 1:** Baseline characteristics of study participants.

Number of patients (*n* = 332)	
**Demography**	
Mean age ± SD (%), *n*	
Women	51.46 ± 11.47, (44.40%), *n* = 152
Men	52.35 ± 11.03, (55.60%), *n* = 180
**Biochemistry**	
Blood glucose (mg/dL)	257.50 ± 85.50
Alanine aminotransferase (UI/L)	31.00 ± 20.66
Aspartate aminotransferase (UI/L)	29.33 ± 18.23
Creatinine (mg/dL)	12.06 ± 0.62
24 h Urinary glucose excretion (g/day)	17 ± 14
**Baseline glucose‐lowering therapies (BGLT)**	
HbA1c % ± SD, *n*	
Naïve	8.23 ± 1.65, *n* = 43
Glibenclamide	12.80 ± 1.66, *n* = 65
Glimepiride	8.20 ± 1.30, *n* = 40
Metformin	9.13 ± 2.12, *n* = 41
Glibenclamide + metformin	10.85 + 2.13, *n* = 45
Insulin	8.60 ± 1.13, *n* = 58
Glimepiride + metformin + insulin	7.60 ± 1.26, *n* = 40

To facilitate data collection and analysis, patients were categorized based on their baseline glycemia, HbA1c levels, and the type of glucose‐lowering therapies (DGLT) they were receiving during the first visit.

#### 2.2.1 Outcomes

- Primary outcomes: Change in fasting blood glucose and HbA1c from baseline to Day 90.
- Secondary outcomes: Changes in urinary glucose excretion, liver enzymes (ALT, AST), serum creatinine, and functional symptom scores.

### 2.3 Statistical Analysis

Quantitative data are presented as the mean ± standard deviation (SD) of n patients. To account for the heterogeneity in baseline antihyperglycemic therapies, patients were stratified into subgroups based on their Baseline Glucose‐Lowering Therapy (BGLT, see Table 1). The primary analysis was the within‐subgroup comparison of parameters (glycemia, HbA1c, etc.) from baseline (Day −1) to each follow‐up time point (Days 30, 60, 90). For each subgroup, a repeated‐measures ANOVA was performed, followed by Dunnett's multiple comparison post‐test for comparisons against baseline. Statistical significance was set at *p* < 0.05. All analyses were performed using GraphPad Prism version 8.0.2 for Windows (San Diego, CA).

## 3 Results

### 3.1 Effect of BSP on Fasting Glycemia and Glycosylated Hemoglobin

The effect of BSP treatment was evaluated by measuring fasting blood glucose concentration in two patient categories based on their baseline fasting blood glucose levels (category 1 (249–150) mg/dL, and category 2 (350–250) mg/dL) on days 0, 30, 60, and 90. The combined administration of BSP and oral antidiabetic medication led to a significant reduction in fasting blood glucose concentration (*p* < 0.001). This reduction in blood glucose was not directly dependent on the category of baseline values of glucose. It was observed that when the baseline fasting blood glucose concentration exceeded 200 mg/dL, BSP reduced glycemia to approximately 125 mg/dL within 1 month. In contrast, when the baseline fasting blood glucose was around 150 mg/dL, the reduction was closer to 100 mg/dL. We also observed that BSP could regulate fasting blood glucose concentration after 1 month in all patient categories. The combined oral administration of BSP and antidiabetic medication was evaluated across different patient categories, based on their baseline fasting blood glucose levels before treatment. The results demonstrate that BSP significantly reduced fasting blood glucose concentration within 1 month (*p* < 0.0001). We also analyzed the effect of BSP on glycosylated hemoglobin (HbA1c) based on patients' baseline glucose‐lowering therapies (BGLT) and their initial HbA1c levels during visit 1, before BSP administration. The effect of BSP was evaluated in patients previously receiving BGLT. All the combinations with BSP resulted in a significant reduction in HbA1c levels, indicating a clinical benefit when used alongside oral antidiabetic drugs. Notably, the combination of BSP with insulin, as well as with insulin plus Glimepiride and Metformin, showed a significant reduction in HbA1c levels (Table 2).

**TABLE 2:** Effect of BSP on fasting glycemia and glycosylated hemoglobin in the 90‐day study, (HbA1c measured at baseline, Day 60, and Day 90).

Variable	BGLT	Category	Day‐1	Day 30	Day 60	Day 90
Glycemia (mg/dL) Mean (SD)		[350–250]	317 (26), *n* = 112	152 (25)^a^, *n* = 112	132 (28)^a^, *n* = 112	98 (11)^a^, *n* = 112
		[250–150]	198 (28), *n* = 220	149 (15)^a^, *n* = 220	129 (17)^a^, *n* = 220	103 (15)^a^, *n* = 220
HbA1c (%) Mean (SD)	Glibenclamide	[13–10]	11.8 (1.6), *n* = 18		6.7 (1.5)^a^, *n* = 18	5.9 (0.8)^a^; *n* = 16
		[10–8]	8.6 (0.7), *n* = 21		5.9 (0.7)^a^, *n* = 21	5.9 (0.5)^a^, *n* = 20
		[8–6]	6.8 (0.4), *n* = 26		6.1 (0.5)^a^, *n* = 26	5.8 (0.8)^a^, *n* = 26
	Glimepiride	[12–7]	8.2 (1.3), *n* = 40		6.2 (0.7)^a^, *n* = 40	5.9 (0.5)^a^, *n* = 40
	Glimepiride + Metformin	[13–7]	10.8 (2.1), *n* = 45		6.7 (1.3)^b^, *n* = 45	6.2 (0.9)^b^, *n* = 43
	Metformin	[13–7]	9.2 (2.1), *n* = 41		6.5 (2.1)^c^, *n* = 41	5.9 (0.6)^b^, *n* = 40
	Insulin	[10–7]	8.6 (1.1), *n* = 58		6.6 (0.2)^a^, *n* = 58	5.8 (0.6)^a^, *n* = 58
	Insulin + Glimepiride + Metformin	[10–7]	7.8 (1.2), *n* = 40		6.4 (0.6)^a^, *n* = 40	5.9 (0.7)^a^, *n* = 40
	Naïve	[10–6]	8.2 (1.6), *n* = 43		5.9 (1.4)^c^, *n* = 43	5.8 (0.6)^c^, *n* = 43

*Note:* HbA1c was measured at baseline (Day −1), Day 60, and Day 90. Blood glucose was measured at all time points. Comparison by category was performed by Dunnett's multiple comparison post‐test using Graph Pad Prism version 8.0.2 for Windows.

Abbreviations: BGLT, baseline glucose‐lowering therapies; HbA1c, glycosylated hemoglobin.

^a^ *p* < 0.0001, ^b^ *p* < 0.001, ^c^ *p* < 0.05.

### 3.2 Effect of BSP on Urinary Glucose Excretion (UGE)

**FIGURE 1:**
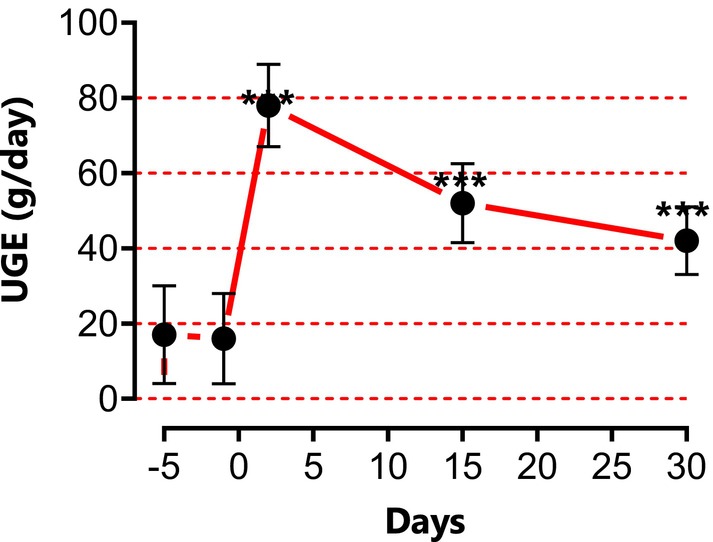
Urine glucose excretion (UGE) estimated by measuring the total amount of glucose in 24‐h urine samples collected on days −5, −1, 2, 15, and 30. ***p < 0.001.

The oral administration of BSP at a dosage of 350 mg three times daily significantly increased urinary glucose excretion (UGE) over the subsequent 24 h (60 g/day; Figure 1). This was accompanied by an increase in urine volume (polyuria) and frequency of urination (pollakiuria), with the most pronounced effects observed during the initial days of treatment (3000 mL and sometimes more). These parameters gradually returned to baseline values by day 15.

### 3.3 Effect of BSP on Serum Aminotransferase Activities and Creatinine

During the clinical trial, serum aminotransferase (ALT, AST) and creatinine levels were monitored to assess whether the combination of BSP and oral antihyperglycemic drugs could affect the function of the liver. The results indicated that in patients with elevated serum aminotransferase levels, the combination of BSP and oral antihyperglycemic drugs significantly reduced hepatic aminotransferase levels to within the normal range. Importantly, this treatment did not affect patients who initially had normal aminotransferase concentrations (Table 3). The combination of BSP with oral antihyperglycemic drugs demonstrated a significant reduction in hepatic alanine aminotransferase (ALT) and aspartate aminotransferase (AST) levels in patients with elevated aminotransferase concentrations. Conversely, BSP did not influence aminotransferase levels in patients who presented with normal concentrations, indicating that BSP may effectively target liver function abnormalities without adversely impacting those with healthy liver enzyme levels.

**TABLE 3:** Effect of BSP on serum aminotransferase activities and creatinine.

Variable	Category	Day‐1	Day 60	Day 90
ALT (UI/L), mean (SD)	[65–40]	54 (7), *n* = 6	32 (6)^c^, *n* = 6	16 (6)^b^, *n* = 6
	[40–20]	25 (6), *n* = 13	20 (5)^ns^, *n* = 13	16 (3)^ns^, *n* = 13
	[20–10]	14 (2), *n* = 30	14 (2)^ns^, *n* = 30	14 (2)^ns^, *n* = 30
ASAT (UI/L), mean (SD)	[55–30]	49 (4), *n* = 8	45 (5)^ns^, *n* = 8	15 (5)^a^, *n* = 8
	[30–20]	26 (5), *n* = 33	24 (6)^ns^, *n* = 33	18 (3)^ns^, *n* = 33
	[20–10]	13 (3), *n* = 11	13 (5)^ns^, *n* = 11	15 (3)^ns^, *n* = 11
CRT (mg/mL), mean (SD)	[15–10]	13 (2), *n* = 37	12 (2)^ns^, *n* = 37	11.4 (2)^ns^, *n* = 37

*Note:* Comparison by category was performed by Dunnett's multiple comparison post‐test using Graph Pad Prism version 8.0.2 for Windows.

Abbreviations: ALT, alanine aminotransferase; ASAT, aspartate aminotransferase; CRT, creatinine.

^a^ *p* < 0.0001, ^b^ *p* < 0.001, ^c^ *p* < 0.05.

### 3.4 Effect of BSP on Functional Adverse Effects Symptoms of T2DM

During the medical visit, various adverse effects and symptoms associated with type 2 diabetes mellitus (T2DM) were evaluated and recorded. These included thirst, vomiting, dry mouth, abdominal pain, impotence, headache, paresthesia, dizziness, nausea, diarrhea, constipation, fatigue, pollakiuria, and urinary frequency (Table 4). At the first endpoint, a significant reduction in the major side effects of diabetes was observed among the patients, indicating an improvement in their overall symptomatology.

**TABLE 4:** The functional adverse effect of T2DM.

Functional symptoms of T2DM	Percentage of adverse effect	
	% Day‐1	% Day‐30
**Gastrointestinal disorders**		
Dry mouth	7.69	0
Thirst	22.22	0
Constipation	3.85	1
Diarrhea	3.85	0
Nausea	14.62	0
Abdominal pain	12.71	0
Vomiting	3.85	0
**Nervous system disorders**		
Paraesthesia	17.69	0
Fatigue	83.1	7
Visual disorders	16,57	3
Dizziness	21,54	0
Headache	11,54	1
**Renal and urinary disorders**		
Impotence	67,82	2
Polyuria	83,75	1
Pollakiuria	35,29	3

## 4 Discussion

The general situation regarding type 2 diabetes mellitus is increasingly concerning. Approximately 422 million individuals worldwide are affected by diabetes, with a significant proportion residing in low‐ and middle‐income countries. Each year, diabetes is directly responsible for an estimated 1.5 million deaths, underscoring the urgent need for effective prevention and management strategies. Both the number of diabetes cases and its prevalence have been steadily increasing over the past few decades. For individuals living with diabetes, access to affordable treatment, including insulin, is essential for their survival and overall well‐being. To address this growing epidemic, there is a globally agreed‐upon target to halt the rise in diabetes and obesity by 2025 (Wold Health Organization 2023). However, antidiabetic medications may not be effective for all individuals with type 2 diabetes, particularly those with inadequate glycemic control or a long duration of the disease. In such cases, clinicians may opt to prescribe a combination of oral antidiabetics that employ different mechanisms of action to enhance glucose regulation. In some cases, oral antidiabetic medications may be used in conjunction with insulin injections, which provide synthetic insulin directly to the body. The choice of medication and dosage is influenced by several factors, including the individual's blood glucose levels, body weight, kidney function, and potential side effects. Among the traditional antidiabetic treatments used in Chad and Cameroon are “lost foods” or famine foods, such as *BS*. The results indicate that BS directly inhibits the intestinal glucose absorption in vitro in mice. Our findings also support the use of BSP in reducing glycemic and HbA1c levels in diabetic patients who are resistant to oral antihyperglycemic medications. Clinical studies have prioritized human trials involving diabetic patients who remain on baseline glucose‐lowering therapies (BGLT) despite resistance to oral antidiabetics. This decision was guided by ethical considerations, as it marked the first instance of clinical studies being conducted on diabetic patients. To ensure the health and safety of our participants while maximizing their chances of achieving normalized blood sugar levels, we opted to incorporate BSP as a therapeutic supplement. Following the confirmation of the positive clinical benefits associated with BSP, we extended the trials to include naive patients with HbA1c level of 8.23 ± 2.68. The clinical studies demonstrated that BSP significantly improved glycemic control in all patients with type 2 diabetes. Notably, A reduction in blood glucose levels was observed after 1 week of treatment. On the other hand, the decrease in HbA1c levels was measurable after 30 days of treatment, remaining below 7% after 60 days of treatment. Additionally, the result indicated that BSP enhanced urinary glucose excretion. More than 300 out of 332 patients reported a significant increase in urine volume (polyuria) and frequency (pollakiuria), particularly notable in the initial days of treatment, with both parameters gradually returning to baseline values by day 15. Weight loss, particularly among overweight individuals, was also observed during treatment with BSP. The enhancement of urinary glucose excretion (UGE) suggests that BS or its active metabolites, Glucocapparin, may act as a dual inhibitor of SGLT1 and SGLT2. This mechanism could be one of the key explanations for its effectiveness in the treatment of type 2 diabetes. Indeed, several antidiabetic agents, including clinically prescribed molecules like canagliflozin, function as dual inhibitors of the sodium‐glucose cotransporters SGLT1 and SGLT2 (Lehmann and Hornby 2016), Empagliflozin (Grempler et al. 2012; Cheng et al. 2016), Ipraglioflozin (Tahara et al. 2012; Komatsu et al. 2020), Luseogliflozin (Kakinuma et al. 2010), and Tofogliflozin (Suzuki et al. 2012). The IC_50_ of BS in mouse intestine was comparable to that of the aforementioned molecules, all of which are derivatives of Phloridzin (phloretin‐2′‐O‐D‐glucopyranoside) (Eto et al. 2024), a naturally occurring bitter metabolite (Tian et al. 2021). For instance, Glucocapparin, a primary methyl glucosinolate component of BS, acts similarly (Eto et al. 2024). SGLT2 inhibitors offer several benefits: (1) they lower blood glucose levels by increasing glucose excretion in the urine, which reduces the risk of severe hypoglycemia; (2) they facilitate glucose removal from the blood without relying on insulin, thereby easing the workload on pancreatic β‐cells; (3) they may contribute to weight loss (4) and they may lower blood pressure by removing excess glucose in the urine. These benefits have been demonstrated in recent studies investigating SGLT2 inhibitors (Musso et al. 2012; Komoroski et al. 2009; Ferrannini et al. 2010; Katsuno et al. 2009). SGLT1 inhibitors reduce glucose absorption in the small intestine and colon and partially decrease renal glucose reabsorption, although this accounts for only about 10% (Zambrowicz et al. 2012). SGLT1 and SGLT2 are sodium‐glucose cotransporters with critical roles in glucose homeostasis, making them promising targets for diabetes treatment. Drugs that inhibit these transporters, such as dapagliflozin, canagliflozin, and empagliflozin, can lower blood glucose levels by reducing intestinal absorption and increasing urinary excretion of glucose. Clinical trials have demonstrated the efficacy of SGLT inhibitors both as monotherapy and as add‐on therapy for type 2 diabetes, offering additional benefits of weight loss and blood pressure reduction (Kosiborod et al. 2017). The main limitations of SGLT inhibitors include their dependence on renal function and their increased risk of urinary and genital infections. Additionally, excessive inhibition of SGLT1 may lead to gastrointestinal side effects. However, SGLT inhibitors operate through an insulin‐independent mechanism, which may offer long‐term glucose control with a low risk of hypoglycemia in type 2 diabetes. They may also play a role in combination therapy with insulin in type 1 diabetes (Tahrani et al. 2013). BSP is associated with weight reduction in both animal studies (Zambrowicz et al. 2012) and personal communications involving humans (Nohya et al. 2022). Reducing glucose absorption reduces the transformation of excess glucose into fat storage, also inhibiting intestinal glucose absorption by blocking SGLT1, which could reduce the absorption of Chylomicrons. This is because chylomicrons, which transport lipids, are absorbed at the intestinal villi. To enter the lymphatic vessels, they must pass through tight junctions between the endothelial cells that form the walls of these vessels, which is facilitated by increased hydrostatic pressure in the villi (McDonald 2018). Increasing the hydrostatic pressure facilitates the passage of chylomicrons by enlarging the openings of the tight junctions. Since glucose stimulates the absorption of sodium and, consequently, water, it is reasonable to conclude that glucose may elevate local hydrostatic pressure and thereby promote lipid absorption. Overall, while observational studies are valuable for generating hypotheses and signals regarding drug effects, they are susceptible to various biases that complicate the determination of causal relationships. For this reason, randomized clinical trials are considered the gold standard for assessing a drug's efficacy and safety, as they minimize many of these biases through randomization. Consequently, observational studies should be interpreted with caution, and their findings must be validated by controlled clinical trials.

## 5 Limitations

This study has several important limitations that must be considered when interpreting the results. First and foremost, the single‐arm, uncontrolled design is a major constraint. While we observed significant improvements in glycemic parameters following the addition of BSP, the absence of a randomized control group (e.g., placebo plus standard care) prevents definitive attribution of these effects solely to BSP. Factors such as enhanced patient adherence due to increased clinical attention (the Hawthorne effect), regression to the mean, or the natural progression of concomitant care cannot be ruled out as contributors to the observed outcomes. Therefore, the efficacy results should be viewed as preliminary and hypothesis‐generating.

Additionally, body mass index (BMI) was not systematically recorded at baseline, limiting our ability to assess weight‐related outcomes.

## 6 Conclusion

According to the ethics committee that authorized these clinical trials. It was strongly recommended that preliminary tests should begin on oral drug‐resistant type 2 diabetes patients to ensure the clinical benefits of the new product (BSP) before continuing with naive patients. Giving a new product to people who are already on a basic course of treatment ensures that it does not put them at risk. This is what we have done in this preliminary study. Our findings suggest that BSP may help reduce glycemia and HbA1c in diabetic patients who are resistant to oral antihyperglycemic drugs, with no significant adverse effects observed. These results highlight BSP's potential as a dual SGLT1/SGLT2 inhibitor, offering a novel mechanism of action in the treatment of type 2 diabetes mellitus (T2DM). However, further validation through controlled clinical trials is necessary to confirm these findings and establish the clinical efficacy of BSP.

## Author Contributions

Conceptualization; B.E., J.M.B., and B.G., and J.‐F.D.; data curation; M.A., A.E., and M.B., formal analysis; B.E., A.O., A.E., R.C.N., M.B., and J.M.B., funding acquisition; M.A., F.N., and A.A.Q., investigation; M.A., A.E., M.B., and B.G., methodology; B.E., J.M.B., R.C.N, B.I., and B.G., project administration; B.E., and J.‐F.D., resources; B.E., and J.M.B., software; B.E., J.M.B., A.O., and B.I, supervision; B.E., J.M.B., and J.F.D, validation; A.E., M.B., and B.I., visualization A.E., M.B., and A.O., writing – original draft; B.E., and J.M.B. Writing – review and editing; B.E., A.O., A.E., R.C.N., A.A.Q., M.B., B.I., B.G.

## Funding

This work was supported and funded by the Deanship of Scientific Research at Imam Mohammad Ibn Saud Islamic University (IMSIU) (grant number IMSIU‐DDRSP2601).

## Consent

Informed consent was obtained from all subjects involved in the study.

## Conflicts of Interest

The authors declare no conflicts of interest.

## Data Availability

The original contributions presented in this study are included in the article. Further inquiries can be directed to the corresponding author.
