# Correction to “Boscisucrophage: A Natural SGLT1/2 Inhibitor From *Boscia senegalensis* for Managing Type 2 Diabetes”

**DOI:** 10.1002/fsn3.71859

**Published:** 2026-05-03

Eto, B. , A. Elrherabi , F. A. Nasr , et al. 2026. “Boscisucrophage: A Natural SGLT1/2 Inhibitor From Boscia senegalensis for Managing Type 2 Diabetes.” Food Science & Nutrition 14, no. 3: e71621. 10.1002/fsn3.71621.41804342 PMC12967618

The affiliation of author Fahd A. Nasr should read as follows: “Biology Department, College of Science, Imam Mohammad Ibn Saud Islamic University (IMSIU), Riyadh, Saudi Arabia.”

We apologize for this error.

